# Predictors of Bullying among Athletes in the Romanian Context

**DOI:** 10.3390/ejihpe13100145

**Published:** 2023-09-26

**Authors:** Florin Nichifor, Andrei-Lucian Marian, Silviu-Mihail Tiţă

**Affiliations:** 1 Faculty of Physical Education and Sport, “Alexandru Ioan Cuza” University of Iasi, Toma Cozma Street, No. 3, 700554 Iasi, Romania; florin.nichifor@uaic.ro; 2 Faculty of Psychology and Educational Sciences, Department for Teacher Training, “Alexandru Ioan Cuza” University of Iasi, Toma Cozma Street, No. 3, 700554 Iasi, Romania; andrei.marian@uaic.ro; 3 Faculty of Economics and Business Administration, Department Management, Marketing and Business Administration, “Alexandru Ioan Cuza” University of Iasi, Carol I Street, No. 22, 700554 Iasi, Romania

**Keywords:** bully/perpetrator behaviour, bully–victim behaviour, pre-competitive emotions, traditional male gender role norms, connections with coaches and teammates

## Abstract

The purpose of this study was to examine the explanatory power of a predictive model of bully/perpetrator behaviour in Romanian athletes, consisting of negative pre-competitive emotions (anxiety, sadness, and anger), perception of male gender normativity, and relationships with coaches and teammates. Additionally, we aimed to explore the mediation effect of bully–victim behaviour on the relationship between athletes’ connections with their coaches and bully/perpetrator behaviour. The current research involved a nonexperimental, cross-sectional design exploring the presence of bully/perpetrator behaviour in Romanian male and female athletes. The quantitative methodology was used to collect and analyse the data obtained. Researchers translated, adapted and pretested the questionnaire set to the Romanian cultural background (SEQ, MAMS, CART-Q, In-group Ties Scale, BSQ) before distributing it to 448 participants. 58.7% were male participants, and 41.3% were female participants. The mean age was 21.15 (SDage = 2.37, range = 18–32). The research was conducted in the first half of 2023. SPSS (V. 20) and Hayes’s PROCESS tool were used to investigate the data. The findings demonstrated that in the case of Romanian male athletes, perception of male gender normativity, anger, and weaker connections with coaches are the most important psychological factors in predicting bully/perpetrator behaviour. In contrast, in the case of Romanian female athletes, only weaker connections with coaches and perception of male gender normativity play an essential role in explaining bully/perpetrator behaviour. Additionally, the study demonstrated that bully–victim behaviour mediates the relationship between athletes’ weaker connections with their coaches and bully/perpetrator behaviour. Athletes’ weaker connections with their coaches lead to experiencing a high level of bully/perpetrator behaviour by stressing bully–victim behaviour, which also contributes to achieving a high level of bully/perpetrator behaviour.

## 1. Introduction

Bullying among young people is widespread in any community, culture, or country worldwide. Influenced by factors such as previous negative experiences, the social environment in which they grow up, and family issues, young people can sometimes unintentionally become either aggressors or victims. This can happen with schoolmates, fellow sports team members, parents, teachers, spectators at competitions, or even ordinary people on the street. From another perspective, in many cases, the adults in the lives of young people, influenced by their daily challenges, may adopt or exhibit challenging behaviours for those around them, significantly impacting the younger generation. We believe that such a phenomenon should be taken very seriously. That is why this study, conducted by a multidisciplinary team comprising a psychologist and psychology professor, a coach and physical education teacher, and an economist specialising in organisational culture, seeks to explore the emotions, attitudes, and behaviours of a sample of students from the Faculties of Physical Education and Sports in North-Eastern Romania concerning the phenomenon of bullying, both within and outside the context of sports activities. Bullying is composed of direct or indirect aggressive acts and typically requires that the nature of the aggression be repetitive, intentionally harmful to an individual, and conducted by an individual with higher power status than the victim [1] and is a complex and heterogeneous phenomenon that directly affects hundreds of millions of people each year [2]. Another definition is “negative actions repeatedly perpetrated by one or more persons against another person who is relatively weaker” [3]. Bullying is also viewed as aggressive goal-directed behaviour that harms another individual within the context of a power imbalance [2]. From another perspective, the phenomenon appears in the form of cognitive effects considered relevant in the school environment that could be transposed in the same context within sports clubs “linked to negative feelings about school and reduced student functioning such as poor mental health outcomes, fear of attending schools, and compromised academic performance, school social justice, and school” [4]. Volk et al. (2014) believed that the phenomenon of bullying manifests itself in sports when there is a lack of clear and well-defined individual and team goals. This frustration often arises due to a range of external influences, including personal values, the athlete’s family, the national sports culture, deficiencies in club management, and a shortage of specialists with diverse skills in areas like technical expertise, tactics, psychology, management, and finance related to sports. In such situations, athletes who are motivated by intrinsic factors and draw strength from their training may find themselves tempted to react and resort to violence [2]. Newman et al. (2021) consider that research on bullying in sports focused on two aspects: the experiences characterised in terms of the frequency of different types of aggressive acts, the amounts to which participants are perpetrators or victims and the types, timing and location of bullying behaviour and subsequent outcomes of this behaviour: poor self-esteem, reduce enjoyment, isolation, negative feelings, poor performance [5,6,7,8]. According to USA Today Sport, coach Jim Foster “made an inappropriate comment regarding a female staff member and spoke negatively about his staff to other staff members [9]. News related to this topic is often found in sports newspapers. Children’s participation in sports activities within various clubs implies that the risk of young people being subjected to abuse is reduced under the careful guidance of coaches. However, bullying can take various forms–overt or subtle, intentional or accidental, and may involve psychological, physical, and sexual abuse. This also includes behaviours like hazing and neglect. Teammates or peers are typically the most common perpetrators of sexual violence, hazing, and bullying [10]. Booth et al. (2023) use the concepts of bullying and banter because, in adolescent community football, bullying acts can be accidental and intentional, demonstrating synergies with micro-inequities. Banter must be viewed as a “joke” or “fun”, which are naturally attributable to micro-affirmations but are also inherently ambiguous terms [11].

The main types of bullying are (i) physical (pushing, shoving, punching, hitting, kicking); (ii) verbal (name calling, banter, threatening, teasing, intimidating, yelling abuse, using put-downs); (iii) psychological (ganging up, preventing a person from going somewhere, taking a person’s possessions, sending hostile or nasty emails or text messages); (iv) socially (excluding, alienating, ignoring, spreading rumours); (v) cyberbullying (messages form social networking, Facebook, Instagram, Tik-Tok, WhatsApp). Behind these behaviours lies an inequality of power (physical, verbal, psychological or social) between a weak victim and a strong aggressor [12]. The effects of this phenomenon in sports are relevant for individual and team performance: (i) a child may bully another for social acceptance, power, or admiration from onlookers [13], influenced by the wrong models they observe around them, on the Internet or TV; (ii) most prominent is a lack of or reduced self-esteem (confidence) [14]; (iii) more bullied youth than non-bullied youth report having headaches, sleep problems, and abdominal pain [15]; (iv) can develop symptoms of internalisation behaviour problems such as depression and social anxiety after prolonged exposure to victimisation [16].

A vital characteristic identified in the research on athletes is that the victims of the phenomenon in many cases are athletes [5], perhaps due to different physical aspects of the victim, race, and way of behaviour, which make them “attractive” to those from other teams or the people around the phenomenon (coaches, supporters), even if the sports phenomenon must be characterised by fair play. The purpose of the article is to identify how the phenomenon of bullying influences athletes. The proposed hypotheses try to respond to the purpose of the research, the following directions being relevant.

According to the Antibullying Policies in Europe report [17] based on OECD that included in the PISA analysis a battery of questions about bullying, the average of students reporting any type of bullying was 18% worldwide, and in Europe, 19.4–23.3% in 2025–2018. In 2018, the Netherlands scored lowest with 2% frequent bullying, and Lithuania scored highest with 23% frequent bullying, with an average of 8.2% frequent bullying. For Romania, the OECD analysis has data only for 2018; the value is 12%, i.e., 146% compared to the European average.

### 1.1. The Relationship between Positive Pre-Competitive Emotions (Excitement and Joy), Negative Pre-Competitive Emotions (Anxiety, Sadness and Anger), Weaker Connections with Their Coaches or Teammates and Bully/Perpetrator Behaviour

Athletes have an additional motivation to make an intense and sustained effort to achieve the goals set with their coaches, driven by a series of intrinsic emotions that are fundamental to performance and closely linked to prosocial motivations. Those who are committed to sports and derive satisfaction from participating possess an internal drive [18]. In addition to motivation, the management of emotions is an essential factor for obtaining significant results because stressful situations are frequent in sports activity, generated mainly from individual or team failures, quickly lead to conflict situations with those around (colleagues, coaches, managers, supporters, etc.) [18,19,20]. Additional motivation generates positive emotions (happiness, hope) and negative emotions, such as anxiety, anger, guilt, fear, and shame [21,22,23,24,25,26]. Beyond measures of depression and anxiety, sports participation is associated with many positive developmental outcomes that may contribute to the protection and enhancement of the psyche, including increased physical and cognitive competence, self-esteem, teamwork, social skills, discipline, responsibility, and empathy [27,28]. Influenced by positive and negative emotions, the human brain can react differently depending on their value and in many cases, there are uncontrolled reactions that can lead to bullying, especially at those ages when the athlete does not have the support of a specialist. In addition, at the micro level, several problems can be highlighted at the individual or sports team level divided by Vveinhardt and Fominiene into two groups: unfair competition among athletes, seeking to push out the unwanted person, and the coach’s behaviour orientated to the search for the most helpful athlete, pushing out unsatisfying candidates [29]. The management of these situations depends in many cases on the goals set for the coach by the sports organisation, which tolerates such behaviours, but also on a series of environmental factors (financial, human, etc.) that lead to one decision or another. From a young age, athletes may perceive these injustices and react towards the coach, who may fear their position within the group will be affected and attempt to remove the athlete from the team. Athletes may also respond toward their colleagues, especially if they believe their selection was based on criteria other than performance. The specific cultural context in which these individuals operate can lead to varying degrees of violent reactions and strained relationships.

Sports competitions carry a significant emotional burden, stemming from the hours of training, the pressure exerted by coaches and parents, and perhaps even from supporters. Additionally, competition can trigger a range of antisocial and aggressive behaviours in the athlete’s mind, as intrinsic factors drive them to believe that winning is a necessity [30], and when an athlete realises that their dreams are not coming true because the competitor or opposing team is better prepared, feelings of frustration and uncontrolled emotional reactions can surface. This may manifest as physically striking the opponent, hurling insults, criticising referees’ decisions, or refusing to heed the coaches’ instructions, among other behaviours.

### 1.2. Traditional Male Gender Role Norms and Bully/Perpetrator Behaviour

Traditionally, sports activity has been seen as a place where boys come together, and the skills acquired in various sports become sources of power and prestige in promoting preferred expressions of masculinity and social status among males. The link between the gender of athletes and bullying often depends on how they are trained and emotionally prepared for sports competitions. The relevant question for football is why boys who are encouraged and learn to use coercion, intimidation, and other aggressive tactics during play are more likely to engage in bullying behaviours off the field than their non-athlete peers [31].

Steinfeldt et al. (2012) try to answer this dilemma by stating that bullying in the case of male athletes is identified with traditional male role norms, which are also related to more permissive attitudes and the social norms of a moral atmosphere that condones bullying and a strong identification with male role norms were positively associated with moral functioning that reflects judgments, intentions, and behaviours that support bullying [32]. David and Brannon (1976) identified four key components of the male role: (i) no “sissy stuff”: the stigma of anything vaguely feminine; (ii) the big wheel: success, status, and the need to be looked up to; (iii) the sturdy oak: a manly air of toughness, confidence and self-reliance; (iv) give “’em hell”: the aura of aggression, violence and daring [33]. The emotional context of the man according to which he conducts his activities is based on descriptive norms and injunctive norms and through which are enforced via perceptions that most men do not talk about their sadness and most men disapprove of talking about their sadness, respectively [34]. The role of gender depends on how the athlete is identified in the role of an athlete by his environment (athletes, friends, schoolmates), a fact that generates a strongly felt internal conflict in his attempt to adhere to society’s expectations regarding masculinity [35].

Several studies have indicated that dominance and aggression are salient components of masculine gender role norms in adolescent boys’ social groups [36,37,38,39,40]. On this dimension, we always have an in-group perspective, which means the male perspective, and an outgroup perspective, meaning the female perspective. In this regard, we wanted to assess both male athletes’ conformity to the endorsement of male role norms and female athletes’ perception of the importance of male athletes’ level of conformity to the endorsement of male role norms.

The transformation of these norms and roles into violence leads to a series of normative risks referring to cultural characteristics such as distance from power [41], between athlete and coach, athlete and non-athlete and sexualised idiocultural traditions (e.g., hazing rituals) and constitutive organisational structure including hierarchies, rewards based on performance and compliance, rules and procedures that limit or restrict consultation and athlete input, and legitimisation of touch [42].

### 1.3. The Relationship between Athletes’ Weaker Connections with Their Coaches or Teammates and Bully–Victim Behaviours

The sports phenomenon involves many emotions from everyone involved: athletes, coaches, managers, and spectators, and not always positive ones such as hope and happiness, but also negative ones that generate desirable behaviour or bullying. Such behaviour manifests itself as interpersonal aggression or violence, taking many different forms, direct physical violence against the person by shoving, hitting, or using psychological violence in the form of name-calling, exclusion, humiliation, and rumour-spreading [29] or behaviour was directed and due to this cause emotional reactions such as anger, disappointment, fear, anxiety, sadness, shame or demotivation [43,44]. The connection with the coach can be an experience: (i) positive relationships based on encouragement and praise, the coaches being supportive and reliable or (ii) negative spotlighting, yelling/scolding/bad attitude/rude, and picking favourites [45,46] or abuse, interpersonal violence, and the broader category of maltreatment [47]. Interpersonal constraints, one of the reasons why athletes give up this activity, are compounded by coaches or parents who push young athletes to win at any cost, pressure them to perform at unachievably high levels, and use negative feedback when the children fail to meet their oftentimes exceedingly high expectations [45,48,49]. In a qualitative study carried out by Newman et al. (2021), we were able to identify that coaches must be aware of the fact that the line between normal and bullying is very thin; a small joke formulated incorrectly can easily be transformed into bullying and banter and the role of sports psychologists is to assist the coaches in establishing the moment when your behaviours, words, gestures cross the line [50]. Stand believes that the phenomenon of bullying arises from a different way of perceiving the relationship between athlete and coach. In many cases, what coaches perceive as appropriate, athletes perceive as inappropriate, and, in fact, bullying [45]. Bullying, when viewed from the perspective of those involved in sports activities, often depends on the level of experience, age, and sporting performance of both the individual being targeted and the one engaging in bullying behaviours. A coach from a background of exceptional athleticism is typically regarded with respect and consideration by those they train. Comments, advice, firmness, or even jokes are generally not viewed as bullying and are unlikely to impact the athlete emotionally. Athletes are often conditioned by the cultural norms specific to their sport, which encourage respect for rules and key figures within the sport. Many athletes have grown up with a deep passion and admiration for such figures, making tolerating occasional lapses in coaching behaviour easier.

### 1.4. Predictive Models of Bullying Behaviour in Romanian Athletes

The sports teams, especially the male ones, are the ideal places where the phenomenon of bullying happens. According to Adler, despite the similarities with bullying, the behaviours of intimidating the opponent or teammate, according to the current definitions, are not considered harassment [51] (for example, the roles of a beginner or veteran in a team, the intimidation tactics being known such as everyone’s place in the locker room, the order in the shower after training or a game, wiping the shoes, singing the band’s anthem, etc.), all being part of the team culture, because everyone has passed through these stages. Few young people, especially the very talented ones, have the strength of character to respond to these challenges of the veterans and in many cases, they quickly become highly respected by the group because, in critical moments, they could bring favourable results through their brilliance. The way you talk on the sports field is only there, and very rarely, we can see open conflicts between the players, maybe only when the tension is very high. An obvious example is hockey, where the level of physical stress is very high, which is why the aggressions are also apparent, only because protected by the specific equipment, the hard hits are cushioned by the protections worn. Still, there are moments when things freeze, and the ice rink turns into a boxing ring. Newman et al. (2022) highlight a different fact in professional football teams, namely that the athletes have the power to speak about the violations or abuses committed only that the central concern of the individuals is to obtain personal interests such as selection in the team [52]. What must be emphasised here is that the phenomenon of bullying exists within teams only for many victims, witnesses, and bystanders of bullying in professional football. It is evident they go through a cost–benefit analysis [53], where the risks of reporting are too significant in terms of further bullying or threats to their position on the team [52].

According to the OECD statistics, the situation in Romania is relevant because 34% of the people who participated in the survey considered that they were bullied in the education system. Hence, the relevance of this type of research is higher. In many cases, the situation in performance sports is different because the pressure of the results correlated with the family’s investments, the rigour of the coaches, and the expectations of the managers and the spectators frequently generate bullying situations. One research realised by the Romanian organisation “Save the Children”, “nearly 50% of students have been victims of bullying in schools, 27% admit to being bullies themselves. 8 out of 10 students have witnessed a bullying situation at school”, or “82% of students (over 4 out of 5) have witnessed bullying situations in their school; nearly three-quarters of students (73%) report witnessing bullying incidents in the classroom”, or “more than a quarter of students (27%) admit that they have been in the position of being the perpetrators of bullying” [54].

### 1.5. The Potential Indirect Effect

As we have mentioned above, there is plenty of evidence of the direct effects of variables on athletes’ bully/perpetrator behaviour. Still, being a complex phenomenon involving multiple explanations, the direct relationships of predictors on bully/perpetrator behaviour cover only one side of the coin. According to the previous scientific literature, several variables appear to increase the occurrence of athletes’ bully/perpetrator behaviour [55]. In this respect, we proposed a mediator model where the relationship between athletes’ connections with their coaches and bully/perpetrator behaviour is mediated by bully–victim behaviour.

## 2. The Objective and Hypotheses of Current Study

The main objective of this study is to examine the explanatory power of a predictive model of bully/perpetrator behaviour in Romanian athletes, consisting of negative pre-competitive emotions (anxiety, sadness, and anger), perception of male gender normativity, and relationships with coaches and teammates. Furthermore, the present research aims to explore the mediation effect of bully–victim behaviour on the relationship between athletes’ connections with their coaches and bully/perpetrator behaviour.

Based on previous research, we hypothesised the following:

**H1.**  *In Romanian athletes, positive pre-competitive emotions (excitement and joy) and weaker connections with their coaches or teammates tend to be negatively related to higher levels of bully/perpetrator behaviour. In contrast, negative pre-competitive emotions (anxiety, sadness, and anger) are positively associated with the bully/perpetrator’s behaviour.*

**H2.**  *Romanian male athletes who adopt traditional male gender role norms are more likely to engage in bully/perpetrator behaviours*.

**H3.**  *Athletes’ weaker connections with their coaches or teammates correlate with higher levels of bully–victim behaviours*.

**H4.**  *Negative pre-competitive emotions (anxiety, sadness, and anger), perception of male gender normativity, and connections with coaches and teammates form a significant predictive model of bullying behaviour in Romanian athletes.*

**H5.**  *There is a mediating effect of bully–victim behaviour on the relationship between athletes’ connections with their coaches and bully/perpetrator behaviour, in that athletes’ connections with their coaches are associated with bully/perpetrator behaviour both directly and indirectly through bully–victim behaviour*.

## 3. Materials and Methods

### 3.1. Research Design

The present study implied a nonexperimental, cross-sectional design exploring some factors that could explain bullying in athletes in the Romanian context. The quantitative methodology was used to collect and analyze the data obtained from all the respondents. Researchers translated, adapted, and pretested the questionnaire set to the Romanian cultural background (Sport Emotion Questionnaire—SEQ, Meanings of Adolescent Masculinity Scale—MAMS, The Coach-Athlete Relationship Questionnaire—CART-Q, In-group Ties Scale and The Bullying in Sport Questionnaire—BSQ) before distributing it to 448 participants. SPSS (V. 20) and Hayes’s PROCESS tool were used to investigate the data.

### 3.2. Population and Sampling

A total of 383 young athletes, students of faculties of Physical Education and Sport from the region of Moldavia, Romania, completed the survey designed to collect information including bullying in sports, emotions in sports, male gender normativity and the relationships with coaches and teammates. The set of questionnaires was accessed online using Google Forms. The convenience sampling technique was used in this study, meaning that only approachable and available athletes were included in the participants group. 58.70% were male participants, and 41.3% were female participants. The mean age of the participants was 21.15 (SDage = 2.37, range = 18–32). All participants signed an informed consent for inclusion before participating in the study. This research was conducted in the first half of 2023 under the Declaration of Helsinki. The protocol was approved in May 2023 by the Ethics Committee of Physical Education and Sport Faculty “Alexandru Ioan Cuza” University of Iasi, Romania.

### 3.3. Measurements

The five concepts in the research design were measured using specific questionnaires pretested on Romanian participants. The subsequent sections provide further information on the reliability of these instruments.

Bullying in sport was measured using the Bullying in Sport Questionnaire (BSQ) developed by Evans et al. (2016), who had adapted the instrument according to Health Behaviours in School-aged Children (HBSC) and Adolescent Peer Relations Instrument (APRI) [56]. This tool covers 36 questions that examine the bullying experience in the sport and school context. Answers were collected on a 5-point Likert scale ranging from 1 (never been bullied) to 5 (bullied several times) to measure the level and types of bullying within sports teams. Additionally, the frequency of bullying victimisation and bullying perpetration were measured. In calculating the internal consistency of the research instrument, Cronbach’s alpha is commonly used to evaluate reliability. As suggested by many authors, a value of 0.70 or higher for Cronbach’s alpha demonstrates the satisfactory reliability of an instrument [57]. To determine the questionnaire’s reliability on the Romanian sample of participants, the SPSS software program (V.20) processed the data. The findings indicated that Cronbach’s alpha for the Bullying in Sport Questionnaire (BSQ) was 0.97.

To measure pre-competitive emotion grounded in the experience of athletes, we used the Sport Emotion Questionnaire (SEQ), developed by Jones, Lane, Bray, Uphill and Catlin (2005) [58]. The scale includes 22 items, rated on a 6-step Likert scale from 0 (“not at all”) to 6 (“extremely”) and assesses anger, anxiety, dejection, excitement, and happiness. On the Romanian sample of participants, Cronbach’s alpha for the Sport Emotion Questionnaire (SEQ) was 0.78.

To assess the endorsement of male role norms among Romanian athletes, we used the Meanings of Adolescent Masculinity Scale (MAMS) by Oransky and Fisher (2009) [59]. The instrument includes 15 items, and subjects had to point out on a scale from 1 to 4 (1—strangle disagree, 4—strongly agree) the extent to which each statement fits them. On the Romanian sample of participants, Cronbach’s alpha for Meanings of the Adolescent Masculinity Scale (MAMS) was 0.86.

Connection to coach was measured by The Coach-Athlete Relationship Questionnaire (CART-Q). The instrument is a 22-item measure developed to assess the nature of the coach-athlete relationship as reported by both coaches and athletes (Jowett and Ntoumanis, 2004) [60]. The CART-Q consists of three subscales, including (a) commitment, (b) closeness, and (c) complementarity. For the purposes of this study, only the athlete-perspective measure was included (11 items). Each item was measured on a 7-point Likert-type scale ranging from 1 (Strongly Disagree) to 7 (Strongly Agree). Cronbach’s alpha for The Coach-Athlete Relationship Questionnaire (CART-Q) on the Romanian sample of participants was 0.95.

To measure connection to teammates, we used The In-group Ties Scale subscale from the Three-Factor Model of Social Identity (Cameron, 2004) [61]. The In-group Ties Scale was the only factor selected for use, as it worked as a proper operational definition (i.e., a participant’s perception of emotional closeness and sense of belonging to a team) for assessing the connection of teammates within a team. Each item was rated on a 7-point Likert-type scale, from 1 (Strongly Disagree) to 7 (Strongly Agree). On the Romanian sample of participants, Cronbach’s alpha for the In-group Ties Scale was 0.96.

### 3.4. Data Analysis

The data were analysed using the SPSS software program, Version 20.0. As mentioned above, a pilot study was carried out to verify the reliability of the instruments on a sample of 30 students. The study was carried out by distributing the questionnaire set to 448 participants. Of 440 filled questionnaires, only 383 responses that passed the SPSS screening and cleaning were included in the current analysis. In the current investigation, hypotheses were formulated to determine whether there were statistically significant relationships between bullying and some variables that have been chosen based on a literature review. As a result, Pearson’s correlation, the Stepwise multiple regression method, and mediation analysis using Hayes’s PROCESS tool are the most appropriate ones to test the hypotheses.

## 4. Results

Firstly, we hypothesised a set of positive relationships between positive pre-competitive emotions (excitement and joy), negative pre-competitive emotions (anxiety, sadness and anger), traditional male gender role norms and bully/perpetrator behaviour. Specifically, we assumed that positive pre-competitive emotions (excitement and joy) and athletes’ weaker connections with their coaches or teammates are negatively related to bully/perpetrator behaviour. In contrast, negative pre-competitive emotions (anxiety, sadness and anger) and endorsement in the athletes of traditional male gender role norms are positively related to bully/perpetrator behaviour. In addition, athletes’ weaker connections with their coaches or teammates are also negatively associated with bully–victim behaviour.

An a priori power analysis was conducted using G*Power version 3.1.9.7 (Faul et al., 2007) [62] to determine the minimum sample size required to test the study hypothesis. Results indicated the required sample size to achieve 80% power for detecting a medium effect, at a significance criterion of α = 0.05, was *N* = 84 for Correlation: Bivariate normal model. Thus, the obtained sample size of *N* = 383 adequately tests the study hypothesis.

Statistical data revealed the existence of a significant negative correlation between joy and bully/perpetrator behaviour, a significant negative correlation between athletes’ weaker connections with their coaches and bully/perpetrator behaviour, and a significant negative correlation between athletes’ weaker connections with their teammates and bully/perpetrator behaviour. This means, on the one side, that young athletes who obtain low scores on joy and, on the other, have a weaker connection with their coaches tend to obtain high scores on bully/perpetrator behaviour.

Our results also indicated the presence of a significant positive correlation between anxiety and bully/perpetrator behaviour, a significant positive correlation between sadness and bully/perpetrator behaviour, a significant positive correlation between anger and bully/perpetrator behaviour, and a significant positive correlation between traditional male gender role norms and bully/perpetrator behaviour. In other words, the higher the level of anxiety, sadness, anger and internalisation of traditional male gender role norms in Romanian athletes, the more likely they are to engage in bully/perpetrator behaviours (Table 1).

Regarding the bully–victim behaviour condition of athletes, the Pearson correlation analysis showed a significant negative association between athletes’ weaker connections with their coaches and bully–victim behaviour and a significant negative association between athletes’ weaker connections with their teammates and bully–victim behaviour. This means that athletes’ weaker connections with their coaches and teammates tend to be related to a high level of bully–victim behaviour (Table 2).

To verify the effectiveness of some explanatory models of bully/perpetrator behaviour in Romanian athletes, based on negative pre-competitive emotions (anxiety, sadness and anger), perception of male gender normativity and connections with coaches and teammates, a Stepwise multiple regression was conducted. The significant values of standardised coefficients and changes in R^2^ were observed to evaluate the power of the interaction terms to explain variance beyond that accounted for by the main effects in the equation.

At step 1 of the analysis, the perception of male gender normativity entered into the regression equation and was significantly related to bully/perpetrator behaviour F (1380) = 28.61, *p* < 0.001. The coefficient of determination R^2^ was 0.07, indicating approximately 7% of the bullies—the perception of male gender normativity scores could account for perpetrator behaviour variance. Connections with coaches entered into the equation at step 2 of the analysis (t = −4.16, *p* < 0.001). Thus, model 2, consisting of perception of male gender normativity and connections with coaches, was also significant F (2379) = 23.58, *p* < 0.001, accounting for 11% of the bully/perpetrator behaviour variation. Adding anger into the equation at step 3, the regression model explained an additional 1% of the variation in bully/perpetrator behaviour, and this change in R^2^ was significant [F (3378) = 17.71, *p* < 0.001]. The results indicated that model 3 (consisting of perception of male gender normativity, connections with coaches, and anger) is the most accurate explanatory model, having a coefficient of determination R^2^ = 0.12, which means that the variables of model 3 account for 12% of bully/perpetrator behaviour variance (Table 3). Anxiety scores (t = 1.21, *p* > 0.05), sadness scores (t = −0.307, *p* > 0.05) and connections with teammates scores (t = −0.319, *p* > 0.05) did not enter into the equation at the next step of the analysis.

Differential analysis of the explanatory power of predictors on bully/perpetrator behaviour according to the gender (male and female) of Romanian athletes has led us to have a different picture. Therefore, the significant regression model consisting of perception of male gender normativity, connections with coaches, and anger obtained on the whole group of Romanian athletes was run only for male athletes, mentioning that anger has a greater explanatory power of bullying in male athletes (Table 4).

Statistical data also revealed that model 3, consisting of the perception of male gender normativity, anger, and connections with coaches, is the most accurate explanatory model, having a coefficient of determination R^2^ = 0.10. It means that approximately 10% of the bully/perpetrator behaviour variance in Romanian male athletes could be accounted for by variable scores entered into the regression equation (Table 4).

In the case of female athletes, our results showed a specificity effect of predictors in explaining bully/perpetrator behaviour. Anger did not play a significant role in the dynamics of bully/perpetrator behaviour anymore as it did for male athletes; the only factors that were significantly associated with bullying in model 2 were connections with coaches and perception of male gender normativity F (2155) = 12.45, *p* < 0.001. Additionally, anxiety scores (t = 0.122, *p* > 0.05), sadness scores (t = −0.536, *p* > 0.05) and connections with teammates scores (t = 0.014, *p* > 0.05) did not enter into the equation at the next step of the analysis. Therefore, model 2, formed by connections with coaches and perception of male gender normativity, is the most accurate explanatory model, having a coefficient of determination R^2^ = 0.14. It means that approximately 14% of the bully/perpetrator behaviour variance in Romanian female athletes could be accounted for by variable scores entered into the regression equation (Table 5).

The explanatory models of bully/perpetrator behaviour we have tested so far have shown a direct relationship perception of male gender normativity, anger, and connections with coaches (as predictors) and bully/perpetrator behaviour (as outcome), but given the complexity of this phenomenon. Based on previous evidence, we can accept that the relationships between the factors involved in the study are multifaceted. So, we hypothesised that the relationship between athletes’ connections with their coaches and bully/perpetrator behaviour is mediated by bully–victim behaviour. This model suggests that the relationship between athletes’ weaker connections with their coaches and bully/perpetrator behaviour is not a direct effect but operates through an increasing bully–victim behaviour. A mediation analysis using Hayes’s PROCESS tool was conducted to examine this hypothesis.

According to statistical data, there was a significant indirect effect of athletes’ connections with their coaches on bully/perpetrator behaviour β = −0.084, BCa CI [−0.154, −0.021] and similar, K^2^ = 0.096, 95% BCa CI [0.027, 0.177]. Given the value of the K^2^ index being between 1 and 2, this indirect effect can be interpreted as representing 9.6% of the maximum possible value, which means that the effect is relatively small. Athletes’ weaker connections with their coaches lead to experiencing a high level of bully/perpetrator behaviour by stressing bully–victim behaviour, which also contributes to achieving a high level of bully/perpetrator behaviour. In addition, Sobel test results showed the existence of partial mediation in the proposed conceptual model (z = −2.66, *p* < 0.05). In conclusion, bully–victim behaviour partially mediates the relationship between athletes’ weaker connections with their coaches and bully/perpetrator behaviour.

**Figure 1:**
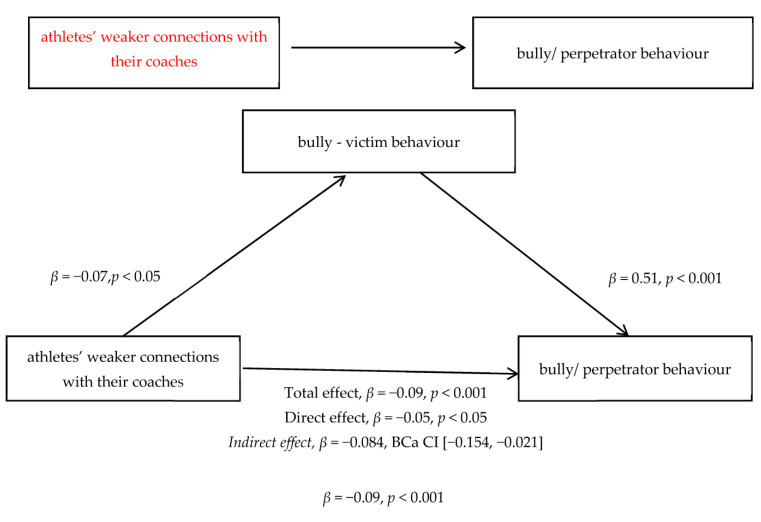
Model of athletes’ connections with their coaches and bully/perpetrator behaviour, mediated by bully–victim behaviour.

As shown in Figure 1, the results indicate the existence of partial mediation in the proposed conceptual model; in other words, bully–victim behaviour partially mediates the relationship between athletes’ weaker connections with their coaches and bully/perpetrator behaviour. These relationships are in the predicted direction.

## 5. Discussion

It is well known that participation in sports is associated with many positive and negative effects. Strictly related to the negative consequences of sport, the current study has explored several psychological factors that facilitate understanding the occurrence of bully/perpetrator behaviour in Romanian athletes. Over the years, researchers have discovered various pieces of evidence that have more clearly articulated the dynamics of bullying in sporting contexts. Similar to other studies [18,19,20,21,22,23,24,25,26,63,64], we found a positive association between negative emotions (anxiety, sadness and anger) and bullying behaviour with regard to the perpetration, meaning that Romanian athletes with high levels of anxiety, sadness and anger will tend to become more involved in bully/perpetrator behaviours [27,28,29]. As well as previous studies [65], we have revealed a significant negative connection between positive emotions like joy and bully/perpetrator behaviour. More specifically, Romanian young athletes who obtain low scores on joy tend to obtain high scores on bully/perpetrator behaviour [31,32]. Another significant result that our study showed concerned the association between traditional gender beliefs and greater involvement in aggressive behaviours, including sports bullying. Our results were consistent with the scientific literature on this topic [31,32,33,34,35,66]. So, we found out that in Romanian athletes, there is a significant positive association between traditional male gender role norms and bully/perpetrator behaviour, which means the higher the level of internalisation of traditional male gender role norms in Romanian athletes, the more they will engage in bully/perpetrator behaviours. In addition, two factors have been documented in the research literature as playing an essential role concerning bullying in sports. It regards the athletes’ relationships with their coaches [43,44,45,46,48,49,67,68] and teammates [69,70]. Considering these facts, our data indicated that Romanian athletes with weaker connections with their coaches and teammates tend to develop bullying behaviour from both victim and perpetrator perspectives.

In the present research, we have attempted to unravel some of the factors that predict the presence of bullying among Romanian athletes. In this multidimensional bullying mechanism, perception of male gender normativity, connections with coaches, and anger played the most crucial role, as together, they explain 12% of bully/perpetrator behaviour variance. Regarding the meaning of these relationships, there was a direct, positive, and significant association between the perception of male gender normativity, anger and bully/perpetrator behaviour. There was also a direct, negative, significant association between connections with coaches and bully/perpetrator behaviour. This means that, as the perception of male gender normativity and anger increase, bully/perpetrator behaviour also increases. The weaker the connections between Romanian athletes and their coaches, the more bully/perpetrator behaviour increases. Hence, compelling evidence shows these are significant predictors of bully/perpetrator behaviour.

According to Hellstrom and Beckman, focus group analysis has one category, “expectations and needs to fit the norm” [71]. Three subcategories: “bullying to achieve power” (girls on their own do not bully other girls, but groups of younger girls bully each other or someone within the group; boys perceive themselves to use violence and to alleviate their aggression physically), “coping with bullying” (the group works as an essential support system when coping with bullying) and “behaviour expectations based on gender. (Boys consider that girls who like activities typically seen as masculine stand out negatively, and girls expect boys to be nice, calm, proper, neat, and to care about their looks)” [72]. Other research considers that the frequent forms of victimisation are insults, followed by physical aggression, more evident for boys and less assumed (talking about the other person). More indirect forms of aggression are more frequent among girls [73].

Based on the paradigm of gender differences in bullying behaviour engagement, which states that males are more likely to engage in–and be victims of–physical bullying in comparison to females [55,73,74,75,76], we tested that thesis on our group of participants (Romanian athletes) to determine whether those earlier results match up. Interestingly, in our study, the data showed a gender difference in favour of male athletes only in the case of bully/perpetrator behaviour but not in the role of bully–victim behaviour, where we found no significant gender difference. Additionally, we have conducted a differential analysis of the explanatory power of predictors on bully/perpetrator behaviour according to the gender of Romanian athletes to have a clearer picture of this phenomenon. The results revealed two different predictive models of bully/perpetrator behaviour by the gender of Romanian athletes. On the one side, when talking about Romanian male athletes, perception of male gender normativity, anger, and connections with coaches provide the most accurate explanatory model of bully/perpetrator behaviour. On the other side, in the case of Romanian female athletes, only connections with coaches and perception of gender normativity (conceptualised as behavioural expectations based on biological sex) significantly predict the bully/perpetrator behaviour.

Looking to identify the conceptual and relational map conducive to bully/perpetrator behaviour, we understood that our explanatory models of bully/perpetrator behaviour, involving a direct effect of predictors on the dependent variable, cover only one side of the coin. Based on previous studies [55,77], we proposed a mediator model where the relationship between athletes’ connections with their coaches and bully/perpetrator behaviour is mediated by bully–victim behaviour. So, we found that the relationship between athletes’ weaker connections with their coaches and bully/perpetrator behaviour is not a direct effect but operates through an increasing bully–victim behaviour, which means that athletes’ weaker connections with their coaches lead to experiencing a high level of bully/perpetrator behaviour by stressing bully–victim behaviour, which also contributes to achieving a high level of bully/perpetrator behaviour.

## 6. Conclusions

The current study provides undeniable evidence about some relevant psychological factors that predict bully/perpetrator behaviour in Romanian athletes and students from the Faculties of Physical Education and Sport in North-Eastern Romania. In this way, we could outline a profile of Romanian athletes in which anxiety, sadness, anger, and traditional male gender role norms positively relate to bully/perpetrator behaviour, while joy and weaker connections with coaches negatively correlate with bully/perpetrator behaviour.

Additionally, the present study has found perception of male gender normativity, connections with coaches, and anger as being the most important predictors of bully/perpetrator behaviour. Putting these constructs in the light of gender differences, we discovered that Romanian male athletes with perceived gender normativity, anger, and connections with coaches are engaged significantly more in bully/perpetrator behaviour in comparison to Romanian female athletes (anger did not play an important role, as much as connections with coaches and perception of gender normativity). As for predictors included in regression equation models, the findings have shown differences in the power explanation of bully/perpetrator behaviour.

The results of this research can help the coach understand and reduce/eliminate bullying actions towards the athlete/team. In sports activities, two types of coaches can be identified: coaches who were athletes and have experienced bullying and coaches who were athletes but have not experienced bullying situations. This study supports both types of coaches, allowing them to learn and understand the phenomenon of bullying and its effects on athletes/teams. There are two possible situations: (i) if coaches experienced bullying as athletes, they can now prevent and reduce this phenomenon among the athletes they train; (ii) if the coach did not experience bullying as athletes, they can now better understand the phenomenon, control it, and mitigate its effects within the team. Knowledge of bullying behaviours (among athletes/teams), understanding this phenomenon, and reducing its impact on vulnerable athletes who may be subjected to bullying can have positive feedback for the athlete/team, manifested via improved performance, increased performance in training/official games, gaining self-confidence, and self-motivation. Furthermore, the coach’s knowledge of the bullying phenomenon (and the results from this study) can help the athlete/team better navigate the challenges of the athlete’s life: training, competitions, and the pressure of the audience, high-level competitions.

Finally, an interesting result that the current research has obtained concerns bully–victim behaviour mediating the relationship between athletes’ connections with their coaches and bully/perpetrator behaviour. Romanian athletes’ significantly lower connection with their coaches has been found to be an essential component of bully/perpetrator experiences by emphasising bully–victim behaviour, which also contributes to achieving a high level of bully/perpetrator behaviour.

## 7. Limitations and Future Research

Even though we started from the premise that previous studies had shown the occurrence of bully/perpetrator behaviour in athletes, the study group that we used (students from the Faculties of Physical Education and Sport in North-Eastern Romania) was the most significant limitation of the current study. The number of research participants was insufficient to cover the variability in responses to the survey questions and generalise conclusions across Romanian student-athletes. Additionally, in terms of methodology, using a Google-form questionnaire could have influenced the objectivity and desirability of the answers to the questions. In this respect, future research could measure study variables by applying the questionnaire set in a face-to-face, paper-and-pencil format to avoid biased responses. Another limitation of the present study was related to adapting the questionnaires to the Romanian cultural setting. Even though the scales showed good psychometric properties, the novelty of exploring bullying in the Romanian sports context using a quantitative methodology could represent a limit of measurement. However, the outcomes of this study have yielded important insights and a broad conclusion regarding possible explanations for nomophobia in young people.

This study opens up opportunities for future research and interpretation to examine the role of specific predictors concerning the different forms of bullying in sports. The current study used an overall bullying score. Still, to better understand bullying mechanisms in athletes, exploring the explanatory power of those factors responsible for developing a particular type of bullying in sport (i.e., physical, verbal, social, and cyber) would be interesting. In addition to the predictors we found in this study to play a significant role in the bully/perpetrator behaviour equation, future research could identify other relevant predictors or mediators.

Finally, future research could investigate the variability of bullying according to the type of sport played: football, basketball, handball, volleyball, and rugby, to see if there is a specificity effect of this behaviour linked to the particular type of sport performed. Although this study found that deficits in the relationship with coaches are an essential component in the predictive model of bully/perpetrator behaviour in Romanian athletes, it was unable to identify the variability in the impact of this relationship by type of sport.

## Figures and Tables

**Table 1:** Correlations between bully/perpetrator behaviour and study variables.

Variables	1	2	3	4	5	6	7	8	9
1. bully -perpetrator behaviour	1	−0.058	−0.115 *	0.106 *	0.113 *	0.157 **	0.265 **	−0.158 **	−0.209 **
2. excitement									
3. joy									
4. anxiety									
5. sadness									
6. anger									
7. traditional male gender role norms									
8. athletes’ weaker connections with their coaches									
9. athletes’ weaker connections with their teammates									

*p* < 0.001 **; *p* < 0.05 * (2-tailed). *N* = 383.

**Table 2:** Correlations between bully–victim behaviour and study variables.

Variables	1	2	3
1. Bully–victim behaviour	1	−0.138 **	−0.130 *
2. Athletes’ weaker connections with their coaches			
3. Athletes’ weaker connections with their teammates			

*p* < 0.001 **; *p* < 0.050 * (2-tailed). N = 383.

**Table 3:** Summary of Stepwise Regression Analysis for variables predicting bully/perpetrator behaviour.

Variable	β	t	sr^2^	R	R^2^	∆R^2^
Step 1				0.26 **	0.07 **	0.07 **
Perception of male gender normativity	0.265	5.34 **	0.07			
Step 2				0.33 **	0.11 **	0.04 **
Perception of male gender normativity	0.259	5.34 **	0.06			
Connections with coaches	−0.20	−4.62 **	−0.04			
Step 3				0.35 *	0.12 *	0.01 *
Perception of male gender normativity	0.251	5.20 **	0.06			
Connections with coaches	−0.187	−3.83 **	−0.03			
Anger	0.114	2.32 **	0.01			

*p* < 0.001 **; *p* < 0.05 * (2-tailed). N = 382.

**Table 4:** Summary of Stepwise Regression Analysis for variables predicting bully/perpetrator behaviour in male athletes.

Variable	β	t	sr^2^	R	R^2^	∆R^2^
Step 1				0.21 **	0.04 **	0.04 **
Perception of male gender normativity	0.216	3.29 **	0.04			
Step 2				0.28 **	0.08 **	0.03 **
Perception of male gender normativity	0.209	3.23 **	0.04			
Anger	0.180	2.78 **	0.03			
Step 3				0.32 *	0.10 *	0.02 *
Perception of male gender normativity	0.199	3.10 **	0.03			
Anger	0.159	2.46 **	0.02			
Connections with coaches	−0.154	−2.37 *	−0.01			

*p* < 0.001 **; *p* < 0.05 * (2-tailed). N = 224.

**Table 5:** Summary of Stepwise Regression Analysis for variables predicting bully/perpetrator behaviour in female athletes.

Variable	β	t	sr^2^	R	R^2^	∆R^2^
Step 1				0.26 **	0.07 **	0.07 **
Connections with coaches	−0.258	−3.34 **	−0.06			
Step 2				0.37 **	0.14 **	0.07 **
Connections with coaches	−0.271	−3.62 **	−0.07			
Perception of male gender normativity	0.268	3.59 **	0.07			

*p* < 0.001 **. N = 158.

## Data Availability

Not applicable.
